# A Meta-Learning Approach for Few-Shot Face Forgery Segmentation and Classification

**DOI:** 10.3390/s23073647

**Published:** 2023-03-31

**Authors:** Yih-Kai Lin, Ting-Yu Yen

**Affiliations:** Department of Computer Science and Artificial Intelligence, National Pingtung University, No. 4-18 Minsheng Road, Pingtung City 90003, Taiwan

**Keywords:** digital forensics, face forgery detection, U-Net, segmentation, meta-learning, few-shot learning

## Abstract

The technology for detecting forged images is good at detecting known forgery methods. It trains neural networks using many original and corresponding forged images created with known methods. However, when encountering unseen forgery methods, the technology performs poorly. Recently, one suggested approach to tackle this problem is to use a hand-crafted generator of forged images to create a range of fake images, which can then be used to train the neural network. However, the aforementioned method has limited detection performance when encountering unseen forging techniques that the hand-craft generator has not accounted for. To overcome the limitations of existing methods, in this paper, we adopt a meta-learning approach to develop a highly adaptive detector for identifying new forging techniques. The proposed method trains a forged image detector using meta-learning techniques, making it possible to fine-tune the detector with only a few new forged samples. The proposed method inputs a small number of the forged images to the detector and enables the detector to adjust its weights based on the statistical features of the input forged images, allowing the detection of forged images with similar characteristics. The proposed method achieves significant improvement in detecting forgery methods, with IoU improvements ranging from 35.4% to 127.2% and AUC improvements ranging from 2.0% to 48.9%, depending on the forgery method. These results show that the proposed method significantly improves detection performance with only a small number of samples and demonstrates better performance compared to current state-of-the-art methods in most scenarios.

## 1. Introduction

In recent years, the issue of forged facial images as a security vulnerability issue has benefited greatly from the development of deep learning. To perform forensic analysis on these types of forged images, deep learning has also been utilized for the purpose of detection. The technology for detecting forged images currently performs well in detecting known methods of forging. This technology uses a large number of original and corresponding forged images created using known forged methods to train the neural networks as the detectors for learning the characteristics of forged images. However, these methods have a significant drop in detection performance when encountering forged methods that have not been trained.

**Figure 1:**
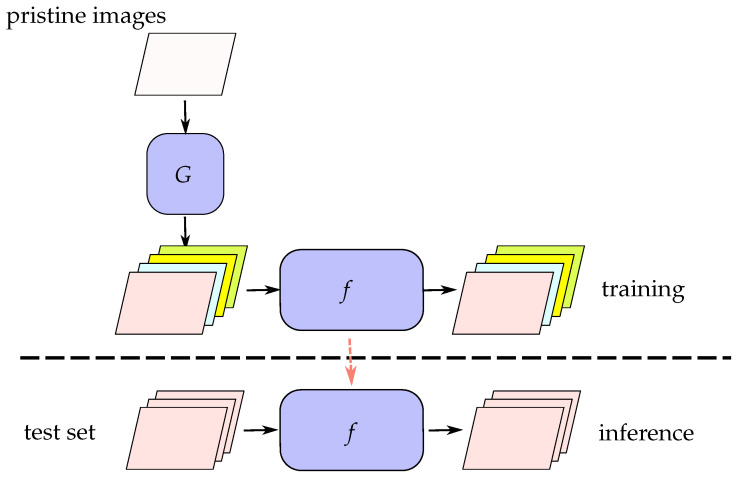
Previous methods of fake sample syntheses. The pristine images are used to generate fake images based on color jitter, resizing, sharpening, and translation using a fake image synthesizer *G*. The orange dashed line represents the use of trained weights during the inference stage.

To address this issue, a novel method has recently been proposed [1]. This method utilizes a parameterizable forged image generator to create diverse forged images, and then uses these forged images to train a neural network. In Figure 1, the pristine image is input to the forged image generator during the training stage. The forged image generator/synthesizer *G* has various mechanisms, such as forging method *A* and forging method *B*, for creating forged images. These synthetic forged images are then used as training images to train the forged image detector. Since the forged image generator is designed by humans based on popular test sets that cover known forging methods, it is equivalent to already seeing forging methods *A* and *B* during the training stage. If the test set contains forging methods that have not been included in the forged image generator, the detector will be unable to effectively detect them. In other words, the aforementioned approach of generating fake images in the training stage cannot exhaust all possible fake mechanisms and, therefore, cannot overcome the problem of encountering fake methods that have not been seen before.

**Figure 2:**
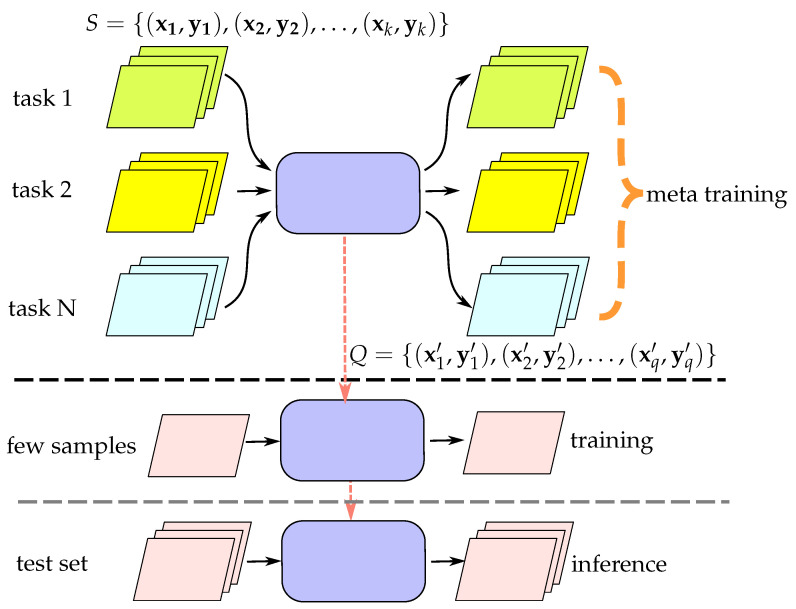
The proposed meta-learning architecture. The orange dashed line represents the flow of weights in the model. During the meta-learning stage, *N* tasks are used to train the model, each with the goal of identifying different forgery techniques. The weights trained during the meta-learning stage are fine-tuned during the fine-tuning stage. Finally, the fine-tuned weights are used for inference.

To address the issue with the methods mentioned earlier, we propose a meta-learning approach to train a detector that excels at identifying new forging techniques. Our method for detecting forged images involves utilizing meta-learning techniques to train a forged image detector that is easy to train with a limited number of new, forged samples. The aim of training the forged image detector is to enable it to take in a small number of new, forged image samples and adjust their weights to identify forged images with comparable statistical features to the provided small set of forged image samples. Please see Figure 2 for a visual representation of this concept. We utilize a collection of forged images generated through various forging methods to construct the training tasks 1 to *N* for meta-learning. The primary objective is not to create a fake image detector that detects all potential forging methods but instead to develop a fake image detector that can be fine-tuned with a few examples of newly developed forging methods.

The primary contributions of this paper can be summarized as follows:

1. Identifying the limitations of existing methods for detecting forged images, particularly in encountering unseen forging techniques;
2. Suggesting a new approach that employs meta-learning techniques to develop a highly adaptive detector for identifying new forging techniques;
3. Proposing a method that fine-tunes the detector, allowing it to adjust its weights based on the statistical features of the input forged images with only a few new forged samples;
4. Showing that the proposed method outperforms current state-of-the-art methods in scenarios where only a few training samples are available.

## 2. Related Work

In the literature, there are various methods for creating fake faces. In this paper, we will specifically introduce a few methods that are relevant to our research. One such method is the NeuralTextures method, as proposed by Thies et al. [2]. This method improves the quality of a computer-generated texture by using a special algorithm in combination with a rendering network to create a realistic reenactment result. Another method, known as the Face2Face facial reenactment system [3,4], maps 2D points of faces to 3D models from source video streams and blends the altered faces from the 3D models with different facial features. A third method, called FaceSwap, is a computer graphics-based approach [5] that extracts facial landmarks of source faces and maps these landmarks onto a 3D template model for creating altered facial features. An additional method for creating fake faces is the deep-learning-based DeepFakes [6] technique. This method involves the extraction of faces from original images, followed by the use of a trained encoder and decoder for the source faces to generate the target fake faces. This technique has been shown to produce highly realistic fake faces and has received significant attention in recent years due to its potential for nefarious usage in the generation of convincing fake videos.

Recent advances in deep learning have been utilized to develop automated methods for detecting fake faces. In the literature, various CNN-based approaches for forgery detection have been proposed [7,8,9]. For instance, Rössler et al. [7] proposed the use of a CNN-based model, namely XceptionNet, to address the forgery detection task as a binary classification problem.

Nguyen et al. [10] proposed a novel approach to the detection of fake faces. They view it as more than just a classification problem, but also as a segmentation problem. In this approach, the focus is on identifying and marking the fake regions of a given image through the use of auto-encoders and a specialized Y-shaped decoder. These techniques utilize information sharing between classification and segmentation for the purpose of detecting and segmenting manipulated facial images. Instead of using the Y-shaped decoder to produce segmentation and classification, the proposed method uses a U-Net model to produce the segmentation result and accords the segmentation result to determine whether the input image is fake.

Zhou and colleagues introduced a groundbreaking architecture called UNet++ in their research [11]. Their approach improves the accuracy of medical image segmentation. The method is founded on a deeply supervised encoder–decoder network that connects the encoder and decoder sub-networks using a series of nested, dense skip pathways. These novel skip pathways aim to reduce the semantic discrepancy between the feature maps of the encoder and decoder sub-networks. The authors postulate that aligning the feature maps semantically facilitates the optimizer to tackle a more manageable learning task.

Feng et al. [12] proposed a new method called MSAK-Net-MCRF for remote sensing multispectral image change detection. MSAK-Net extends U-Net and uses weight-sharing bilateral encoding to extract independent features without adding parameters. SCKB and UM are embedded to extract multi-scale features and express change information. MCRF is used to smooth detection results.

The detection of discontinuities in facial poses between frames within a given video is a method of identifying forged videos. This technique has been extensively studied in the literature [13,14,15], with various studies examining techniques such as chrome-key compositions, duplicated frames, copy-move manipulations, and dropped frames to detect manipulated videos. However, this method of utilizing discontinuities between frames to identify fake videos cannot be applied to identify fake images. Conversely, methods for determining fake images can be utilized to identify fake videos. An illustration of a frame-based methodology is the extraction of frames from videos, which is then followed by the detection of fake frames on a per-frame basis. The fakeness of videos is then determined based on the percentage of fake frames present. For instance, if over 30% of the frames within a video are determined to be fake images, the video can be judged as forged. Thus, the proposed method, which is capable of determining the authenticity of each frame of a video, can also be applied to detect the forgery of videos.

Shiohara et al. [1] proposed a novel approach for detecting deepfake images by utilizing synthetic training data. The method involves using single pristine images as a basis for generating training data, mimicking common forgery techniques. The generated images are designed to be difficult to identify as forged and are used to train a classifier that can distinguish between real and fake images. The method for generating a forged image is to input a pristine image and transform its color and frequency domain features and then use the image as a generated new image. The new image and the original image are used as the source and target images, respectively. Next, a mask is generated, and the source and target images are blended with this mask to obtain a synthetic training image. Their method demonstrates strong performance on a variety of datasets, including FaceForensics++ [7], Celeb-DF [16], DeepFakeDetection [17], DeepFakeDetectionChallenge [18], and FFIW-10K [19]. Notably, the parameters used in the synthetic training image generation process, such as image random resize, mask deform, and blending ratio, are fixed across all test sets. This suggests that the fake image synthesis mechanism employed in their method effectively mimics the forgery methods commonly used in these datasets.

Unlike creating a general detector, another approach based on few-shot learning uses a small number of fake samples to infer unseen forgery methods. The necessity of few-shot learning arises from the fact that, in comparison to human learning, current machine learning algorithms require a significant amount of training data. Few-shot learning [20] is a sub-area of machine learning that attempts to reduce the training set size, with the goal of training with only a few samples to achieve good performance. Korshunov et al. [21] use few-shot tuning on the test dataset to overcome the problem of the accuracy dropping significantly when the model is tested on an unseen dataset.

Meta-learning is one of the methods for achieving few-shot learning. Methods including meta-learning [22], embedders [23], and pre-train models [24] are used to achieve this goal of training with only a few samples. An LSTM-based meta-learner has been applied to learn the classifier problem [20]. They use a meta-learner to capture long-term knowledge common among the tasks. Flennerhag et al. [25] proposed an algorithm that bootstraps a target from the meta-learner and minimizes the distance between meta-learner and target. A method called model-agnostic meta-learning (MAML) [26] adopts a unified training approach: the parameters of the training algorithm are learned by stochastic gradient descent (SGD). This approach to tuning parameters of a gradient-based update meta-learner over the distribution of tasks is widely used to realize few-shot learning [27,28,29,30,31,32,33]. The MAML-like approaches, compared to the meta-learner LSTM, are simpler and easier to train. The proposed method is inspired by the MAML-like approach and aims to learn a model that can easily learn from a small number of fake images to detect unseen forgery methods.

Zhang et al. [34] proposed a Gia-CFSL framework for cross-scene hyperspectral image classification, which combines few-shot learning with domain alignment based on graph information aggregation. The framework addresses the issue of reduced classification performance when there are new classes in target data. Feature-level and distribution-level cross-domain graph alignments are used to mitigate the impact of a domain shift on few-shot learning.

Gao et al. [35] designed new cross-category level vision regression tasks and evaluated common meta-learning techniques on them, providing insights and recommendations for training meta-learning algorithms on vision regression tasks. Additionally, the authors proposed the addition of functional contrastive learning (FCL) over the task representations in Conditional Neural Processes (CNPs) and showed that CNPs outperform MAML on most tasks without fine-tuning.

## 3. The Proposed Scheme

### 3.1. Segmentation-Based Forgery Detection

Given a pristine image t(i,j), where 1≤i≤w and 1≤j≤h, *w* is the width, and *h* is the height of the image, a fake image x is produced by a forgery method g(·). The image t(i,j) can be denoted by matrix notation

$$t=\left[\begin{array}{cccc} t_{11} & t_{12} & \ldots & t_{1w}\\ t_{21} & t_{22} & \ldots & t_{2w}\\ \vdots & \vdots & \ddots & \vdots \\ t_{h1} & t_{h2} & \ldots & t_{hw} \end{array}\right]$$

That is, $t_{ij}=t\left(i,j\right)$. For a color image, each pixel $t_{ij}=\left[r,g,b\right]$ contains three components where r,g,b∈R is the intensity of channels R, G, and B, respectively. This matrix notation is applicable to all images in this paper. The coordinates of the modified pixels are indicated by mask m(i,j), where 1≤i≤w and 1≤j≤h. If $m_{ij}=1$, the pixel $t_{ij}$ of the pristine image is modified by g(·) to produce the pixel $x_{ij}=g\left(t_{ij}\right)$ of the fake image. Otherwise, when $m_{ij}=0$, the pixel $x_{ij}$ remains the same as $t_{ij}$. Given an image x, the segmentation version of the forgery detection problem is to find a matrix $\hat{m}$ such that the difference between ground truth m and predicted $\hat{m}$ is minimal. There are several metrics (e.g., Jaccard similarity coefficient [36], Hausdorff distance [37], and pixel-wise accuracy) to evaluate the difference between m and $\hat{m}$. In this paper, we consider a special case of the problem where the forged area m is a subset of the human face area. The decision version of the forgery detection problem is given an image x to decide if there exists a set of modified coordinates m.

### 3.2. Architecture of the Model

Before delving into the details of our methodology, we first outline the core concept underlying our approach. We use U-Net to identify the forged area in the input image. U-Net can be viewed as a function $f_{\phi }$ with parameters ϕ. To enable U-Net to be trained with a small number of samples, we utilize meta-learning to adjust its parameters.

The information regarding the characteristics of forgery is extracted by optimizing the weights ϕ of the U-Net using the gradient descent algorithm in lines 7–9 of Algorithm 1, which is used to minimize the loss function σ. The parameters of U-Net that are suitable for learning to detect various types of forgery methods with a small amount of data are determined by the outer loop (lines 3–11) of Algorithm 1.
**Algorithm 1:** FakeFaceMetaLearning ($T_{1},T_{2},\ldots ,T_{N}$)**input:** A set of *N* fake fake segmentation task $T_{1},T_{2},\ldots ,T_{N}$**input:** Learning hyperparameters (learning rates) ζ, η**output:** An optimized initial model $f_{\phi }$1 randomly initialize ϕ2 **while** not done **do**   ▹ gradient descent for optimizing ϕ3      **for**  i←1 **to** *N* **do**4          Sample *k* images and their ground truth from support set           $S=\{\left(x_{1},y_{1}\right),\left(x_{2},y_{2}\right),\ldots ,\left(x_{k},y_{k}\right)\}$5          Sample *q* images and their ground truth from query set           $Q=\{\left(x_{1}^{\prime },y_{1}^{\prime }\right),\left(x_{2}^{\prime },y_{2}^{\prime }\right),\ldots ,\left(x_{q}^{\prime },y_{q}^{\prime }\right)\}$6          $\theta_{i}\leftarrow \phi$ ▹ set initialization weight for each task7          **while** not done **do** ▹ gradient descent for optimizing θ8              Evaluate $\nabla_{\theta_{i}}L_{}\left(f_{\theta_{i}}\left(x_{j}\right),y_{j}\right)$ for 1≤j≤k9              Update $\theta_{i}\leftarrow \theta_{i}-\zeta \nabla_{\theta_{i}}L_{}\left(f_{\theta_{i}}\left(x_{j}\right)\right)$ for 1≤j≤k10          $\ell_{i}\leftarrow L_{}\left(f_{\theta_{i}}\left(x_{j}^{\prime }\right),y_{j}^{\prime }\right)$ for 1≤j≤q ▹ count loss using query set11      Update $\phi \leftarrow \phi -\eta \nabla_{\phi }\frac{1}{N}\sum_{i=1}^{N}\ell_{i}$*end*

**Figure 3:**
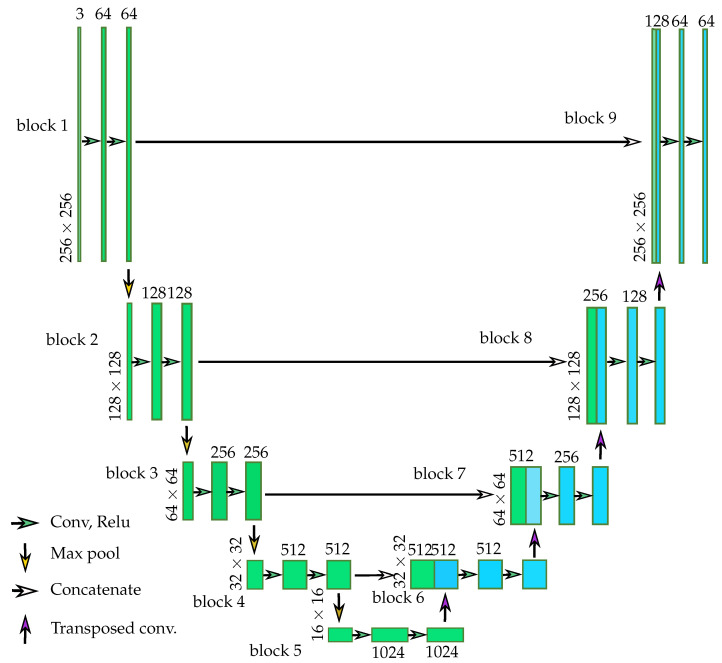
The architecture of the U-Net used in the proposed method. The network has 3 convolutional layers followed by 2 fully connected layers.

To produce the segmentation of predicted manipulated regions, we use the U-Net architecture, a variant of a fully convolutional network, to accept an input image and predict the possibility of the fakeness of every pixel in the input image. The U-Net consists of a sequence of convolutional blocks and transposed convolutional blocks. Figure 3 shows the detailed architecture of U-Net used in the proposed method. The input image x, which contains only RGB channels, is sent to the U-Net, and the predicted mask $\hat{m}$, which only contains one channel, is produced. The input images are first resized to 256×256, and the value of each pixel is normalized with a mean of 0.5 and a standard deviation of 0.5 for each R, G, and B channel. That is, the output of each channel equals the input of the channel minus the mean of the channel divided by the standard deviation of the channel. Afterward, the normalized 256×256×3 images are sent to be the input of U-Net. The sequence of convolutional blocks of U-Net is used to extract the feature of fakeness, and the concatenated sequence of the transposed convolutional block is used to synthesize the predicted mask $\hat{m}$.

For each altered image, the training set also contains its altered area. This altered area, also called a mask, is used to indicate the pixels being modified in the forging procedure. Thus, the mask of an altered image is used as the ground truth of the forged area prediction problem (also called the segmentation problem). Because the images in the dataset include not only facial features but also large areas of background, we use the mask to identify the location of the face in the image, and the nearby area of this location has been cut to be the cropped face. If face detection is used to locate the face in the image and to crop the face part of the image, there will be a failure due to the face detection algorithm being unable to detect the forged image. The cropped images are resized to 256×256 and normalized to a mean of 0.5 and a standard deviation of 0.5. The rectangular area centered on the face in the original image is cropped as the input image of U-Net.

### 3.3. The Meta-Learning Approach

A fake segmentation task trains the model to predict altered pixels of input images using a training set generated by a specific forged method. There are *N* fake segmentation tasks used to train the model, which can easily be adjusted to unseen forgery methods. See the for loop of lines 3–10 in the Algorithm 1. The goal of the algorithm is to find the parameters ϕ that can be trained using only a few samples to detect fake images of unseen methods. When training on task *i*, the inner loop of the meta-learning in lines 7–9 uses gradient descent to adjust the weights, $\theta_{i}$, of the model with one or a few iterations using the support set, $S=\{\left(x_{1},y_{1}\right),\left(x_{2},y_{2}\right),\ldots ,\left(x_{k},y_{k}\right)\}$, which is randomly sampled from the dataset of the task. After completing the whole loop in lines 7–9, line 10 calculates the loss $\ell_{i}=L_{}\left(f_{\theta_{i}}\left(x_{j}^{\prime }\right),y_{j}^{\prime }\right)$ on the query set $Q=\{\left(x_{1}^{\prime },y_{1}^{\prime }\right),\left(x_{2}^{\prime },y_{2}^{\prime }\right),\ldots ,\left(x_{q}^{\prime },y_{q}^{\prime }\right)\}$ using the current weights $\theta_{i}$. After lines 4–10 complete the training of *N* tasks, line 11 uses the average of all the loss $\ell_{i}$ from the tasks to update parameters ϕ using gradient descent. This entire process is repeated until ϕ satisfies the loss requirements. Meta-training is completed up to this point; the process now enters the few-shot learning stage.

In the few-shot learning stage, the optimized model obtained from the previous stage is fine-tuned by *K* fake images from an unseen forgery method *U* using standard gradient descent optimization. After training is finished, we test the model using forged images produced from forgery method *U*.

For simplicity, here, we define the loss function using the notation of two one-dimensional vectors $\hat{y}={\hat{y}}_{1},{\hat{y}}_{2},\ldots ,{\hat{y}}_{n}$, and $y=y_{1},y_{2},\ldots ,y_{n}$. This definition is easy to generalize to higher-dimensional arrays.

(1) $$L\left(\hat{y},y\right)=\frac{1}{n}\cdot \sum_{i=1}^{n}\left(-y_{i}\times \log\left(\sigma \left(\hat{y_{i}}\right)\right)-\left(1-y_{i}\right)\times \log\left(1-\sigma \left(\hat{y_{i}}\right)\right)\right)$$

where $\hat{y}$ is the prediction, and y is the ground truth, e.g., the output of U-Net $f_{\phi }\left(x\right)=\hat{y}$. The logistic sigmoid function σ(·) is defined by $\sigma \left(x\right)=\frac{1}{1+e^{x}}$.

The function σ(·) is utilized in order to introduce nonlinearity into the output of the neurons. This function serves the additional purpose of constraining the output range of the neurons between the values of 1 and 0, thereby enabling the interpretation of the output as a probability of confidence in predicting the fakeness of pixels. Furthermore, σ(·) is differentiable, and this property is of great importance in the Algorithm 1 used to calculate the derivative of the function. This derivative is then employed to update the weights of the U-Net $f_{\phi }$, with the objective of minimizing the error between the predicted and true outputs.

The basic idea behind Formula (1) is to measure the difference between the predicted probability distribution and the true probability distribution of the segmentation (binary classification of pixels). Specifically, the loss function is calculated as the negative log-likelihood of the true class given the predicted probability distribution of segmentation. Intuitively, the loss function measures how well the predicted distribution matches the true distribution of segmentation. If the predicted distribution is very different from the true distribution of segmentation, then the loss will be high, indicating a large amount of uncertainty or disorder in the prediction. If the predicted distribution of segmentation is very similar to the true distribution of segmentation, then the loss will be low.

## 4. Experiment and Comparison

### 4.1. Experimental Design and Data Collection

In order to verify the performance of the proposed method, images altered by the methods DeepFakes, Face2Face, FaceSwap, and NeuralTextures obtained from the FaceForensics++ dataset were used for the experiments. Our experiment involved mixing real images that had not been tampered with and fake images that had been forged using four methods—DeepFakes, Face2Face, FaceSwap, and NeuralTextures—and then feeding the resulting images into the proposed detector. In each experiment, the ratio of forged images to real images is fifty-fifty. This resulting mixed set of images is input into the detector we proposed to detect the forged regions and determine whether the image is fake. As this dataset FaceForensics++ has also been used by other related studies to evaluate the detection capability of their algorithms and their ability to detect forged images, we can compare our results with theirs based on the use of the same dataset.

We utilized the C23 version images from the FaceForensics++ dataset, which was compressed using H264 with a constant rate quantization parameter equal to 23. The purpose of using C23 images is to simulate real-world situations where manipulated images might have their quality reduced by compression or other factors. If a high compression ratio, such as c40, is used, the image will become very blurry. Although it is difficult to determine if the image is forged, such a blurry image cannot be used in general situations. The FaceForensics++ dataset comprises 1000 pristine videos that were obtained from YouTube by the dataset’s creators. The FaceForensics++ dataset is produced from 1000 pristine videos. As illustrated in Figure 3 of the paper [7], there exists a slightly higher number of female characters in these videos when compared to male characters. The dataset encompasses 1.5 million frames that are extracted from the aforementioned manipulated videos. However, we only use 10 frames for each video in this paper. Thus, there are 10,000 fake images for each type of forgery method.

To align our experiments with those previously conducted in [7,10], we utilized the same training and testing set partition. Specifically, videos 0–719 were designated as the training set (also the support set in this paper) and videos 860–999 were designated as the testing set (also the query set in this paper). Notably, a fixed number of training epochs is utilized instead of a validation set to determine when to halt the training process.

The experiments conducted in this paper followed a specific protocol, whereby the training sets used during the meta-training phase were excluded from the inference stage. For example, in Table 1, when the testing set is DeepFakes, the corresponding model under evaluation was not trained on Deepfakes during the meta-training stage.

All experiments were performed on a computer with an AMD 2.23 GHz Ryzen 9 CPU with cache size 512 KB and a Nvidia RTX 3090 GPU for evaluating the performance of the proposed meta-learning approach fake face segmentation and detection. The meta-learning training process, which requires a great deal of computation, benefits from the parallel computing capability of GPU to optimize the detection model.

At the meta-learning stage, we use K=16 for inner-loop training, and the batch size of the query set is 8 for validation. The learning rate of the inner loop is ζ=0.001, and the learning rate η used in line 11 of the Algorithm 1 is 0.003. In the few-shot learning stage, the learning rate is fixed at 0.0001 and the values of *K* are set to 1, 2, 3, 4, 5, 6, 7, 8, 9, 10, 20, 30, 40, 50, 60, 70, 80, 90, 100, 200, 300, 700. During the meta-learning stage, we fix the number of episodes at 6000, perform a single-step inner loop, and use a batch size of 2. In the fine-tuning stage that follows, we set the batch size to 16 and set the epoch to 900. An ‘episode’ represents a complete training iteration on all training tasks, while an ‘epoch’ refers to processing the entire fine-tune set.

**Figure 4:**
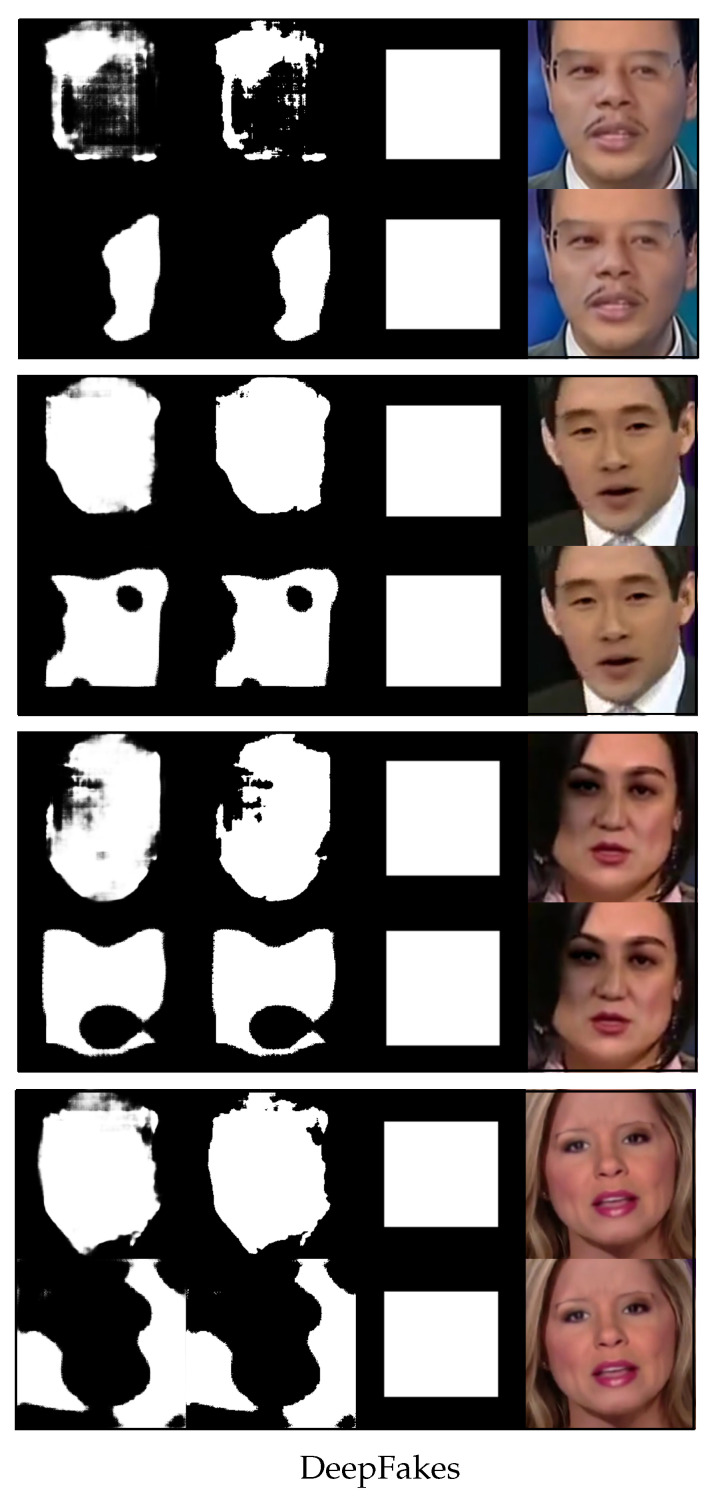
Four rows of randomly selected images from DeepFakes and their predicted results for the fake region with one-shot fine-tuning. The top sub-row of each row is the result generated by the proposed method, and the bottom sub-row of each row is the result without using the proposed method. In each sub-row, from right to left, there is the image altered by DeepFakes, the ground truth of the altered region (mask), the binary predicted output, and the gray-scale predicted output. The results are performed with K=1 shot training.

**Figure 5:**
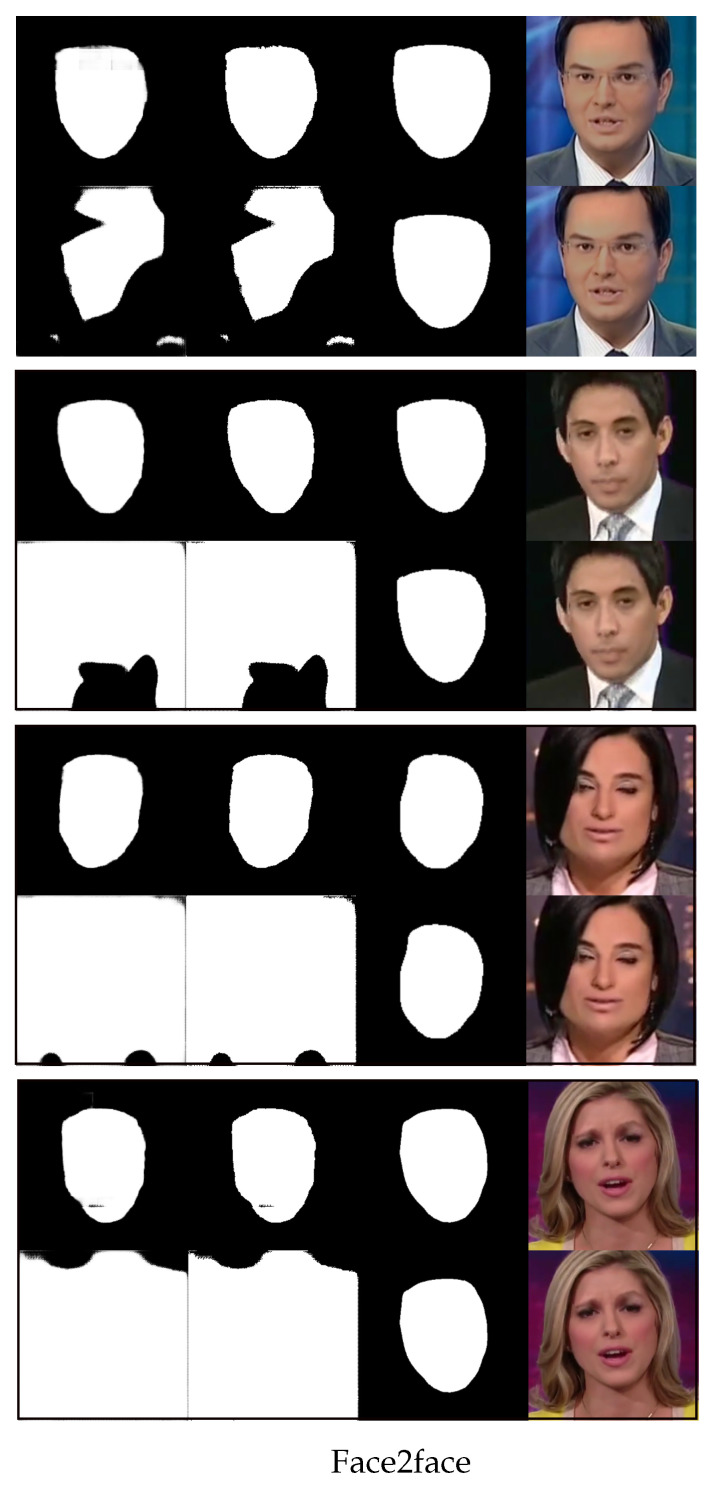
Four rows of randomly selected images from Face2Face and their predicted results for the fake region with one-shot fine-tuning. The top sub-row of each row is the result generated by the proposed method, and the bottom sub-row of each row is the result without using the proposed method. In each sub-row, from right to left, there is the image altered by Face2Face, the ground truth of the altered region (mask), the binary predicted output, and the gray-scale predicted output.

**Figure 6:**
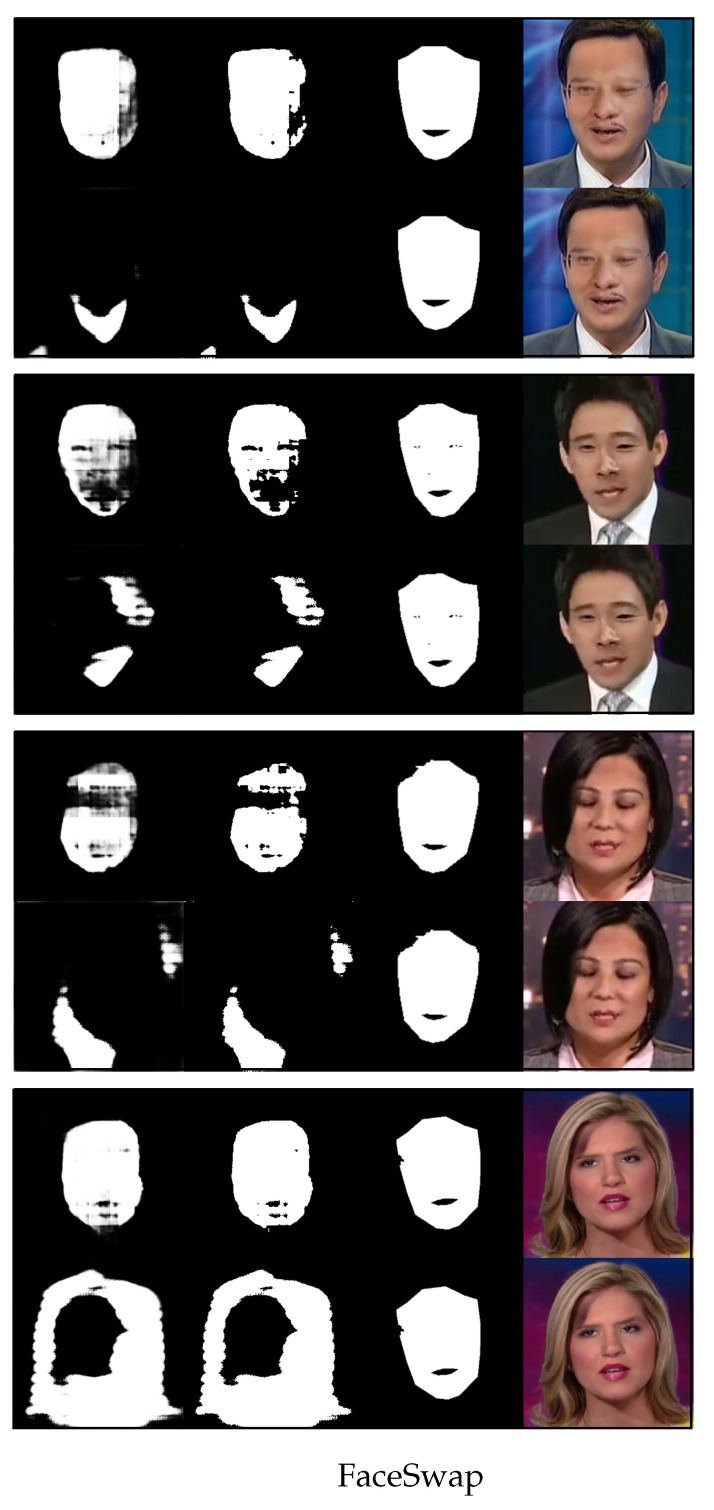
Four rows of randomly selected images from FaceSwap and their predicted results for the fake region with one-shot fine-tuning. The top sub-row of each row is the result generated by the proposed method, and the bottom sub-row of each row is the result without using the proposed method. In each sub-row, from right to left, there is the image altered by FaceSwap, the ground truth of the altered region (mask), the binary predicted output, and the gray-scale predicted output.

**Figure 7:**
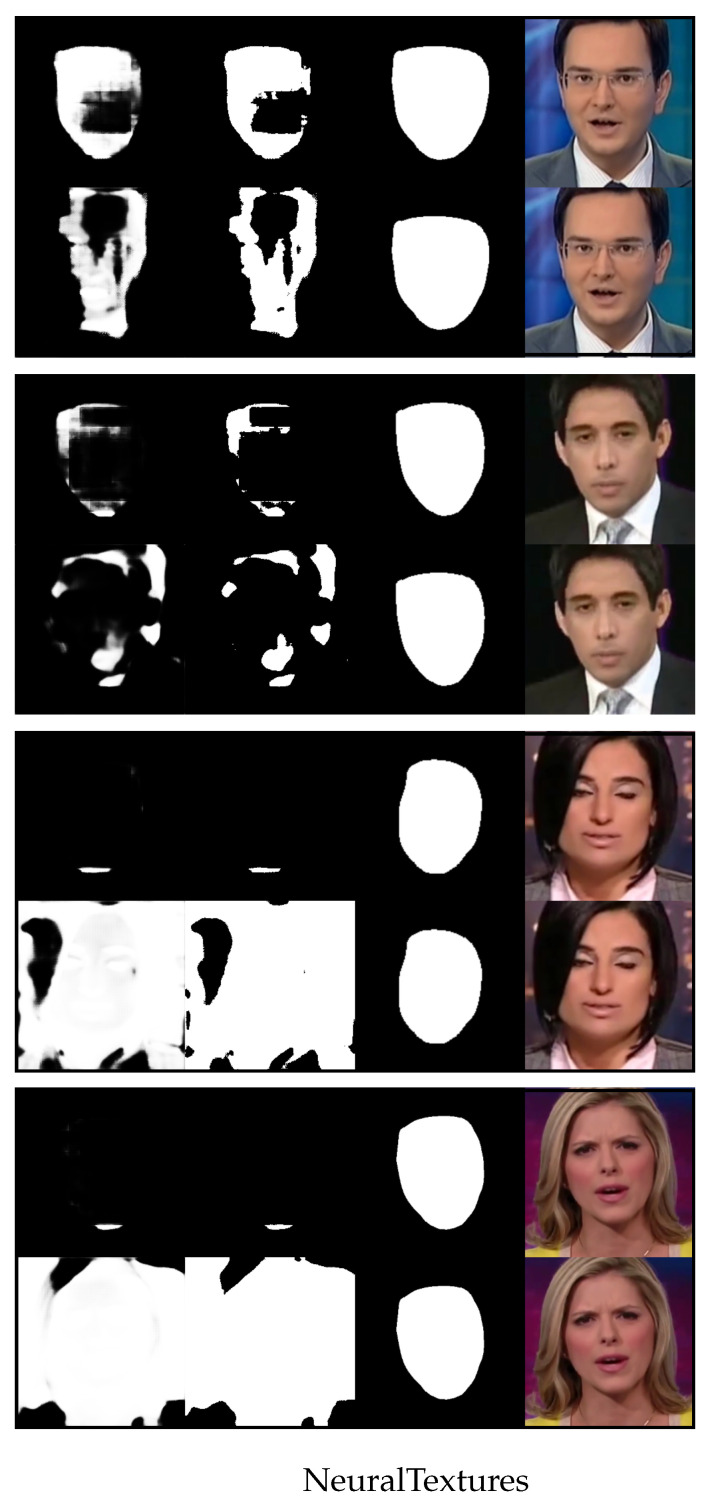
Four rows of randomly selected images from NeuralTextures and their predicted results for the fake region with one-shot fine-tuning. The top sub-row of each row is the result generated by the proposed method, and the bottom sub-row of each row is the result without using the proposed method. In each sub-row, from right to left, there is the image altered by Face2Face, the ground truth of the altered region (mask), the binary predicted output, and the gray-scale predicted output.

We now use Figure 4, Figure 5, Figure 6 and Figure 7 to demonstrate the results of using the proposed method versus not using it to detect four different types of image forgeries. Of the four forgery methods, the proposed method shows the best results in detecting the Face2Face method, while the least effective detection is for NeuralTextures. However, all detection methods outperform the results obtained without using the proposed approach.

Figure 4 shows randomly selected images from DeepFakes and their predicted segmentation results with one-shot fine-tuning. For each row, the top sub-row shows the results obtained using the proposed method, while the bottom sub-row displays the results without its use. Within each sub-row, the images altered by DeepFakes appear on the most right, followed by the ground truth of the altered region (mask), the binary predicted output, and the gray-scale predicted output, moving from right to left.

As depicted in Figure 4, the proposed method results in more accurate segmentation, as demonstrated by a better alignment between the predicted segmentation area and the forgery face area. Although models trained without the use of meta-learning methods are able to learn some of the edges and corners in the forged region, there are many incomplete areas left within the forged region. Due to variations in individuals’ ability to directly judge the boundaries of regions from gray-scale images, in Figure 4, Figure 5, Figure 6 and Figure 7, we aim to present not only the gray-scale images of the system’s raw output but also the binary images after binarization. The percentage of false pixels identified in the binary images is used as the basis for determining whether the entire image is a fake image. With this binary image, the reader can observe how the algorithm classifies real and fake images from its perspective.

Figure 5 shows randomly selected images from Face2Face and their predicted segmentation results with one-shot fine-tuning. The organization of rows and sub-rows in Figure 5 is similar to that in Figure 4. To avoid redundancy, for the format of the figure refer to Figure 4. From Figure 5, we can observe that the proposed method significantly outperforms the method using random initial weights in the test set Face2Face.

The proposed method generates segmentations that are more aligned with the ground truth, while the results produced without using the meta-learning approach exhibit a noticeable deviation from the ground truth. This excellent performance on Face2Face is a surprising result because it is particularly hard to detect forgery technique on Face2Face in other literature [7].

Figure 6 shows randomly selected images from FaceSwap and their predicted segmentation results with one-shot fine-tuning. The organization of rows and sub-rows in Figure 6 is similar to that in Figure 4. As shown in Figure 6, the proposed method exhibits excellent performance in detecting forgeries produced by the FaceSwap technique. The difference between the predicted fake region of the proposed method and the ground truth region is small, while the predicted fake region obtained by the model without meta-learning is fragmented and incomplete. Although our method’s ability to detect the fake regions of FaceSwap forgeries is not as good as that of detecting Face2Face forgeries, it is still very close to the actual fake region.

Figure 7 shows randomly selected images from NeuralTextures and their predicted segmentation results with one-shot fine-tuning. The organization of rows and sub-rows in Figure 7 is similar to that in Figure 4. As shown in Figure 7, the results of detecting the forgery method NeuralTextures using our method are only slightly better than the results without using our method. Our method shows results that are close to the ground truth in row 1, but there is a significant discrepancy between the results in rows 2, 3, and 4 and the ground truth regions. In Figure 4 of article [7], of the paper [7], there exists a slightly higher number of female characters in these videos it can also be observed that the forgery method NeuralTextures is more difficult to detect than Face2Face, making it the hardest to detect among the four methods tested. Their result is consistent with our experimental results in Figure 7.

### 4.2. Performance Metrics

In addition to the qualitative inspection of the quality of the predicted fake region, we also quantitatively inspected the quality of the predicted fake region and measured the performance of determining whether it is a fake image. In Table 1, we use the metric intersection of union (IoU) to measure the quality of the predicted forgery region, and we use the metric area under the receiver operating characteristic curve (AUC) and the metric accuracy (acc) to measure the efficiency of determining whether it is a forged image. If the percentage of the area of an image predicted to be a forgery area reaches a threshold value, the image is judged as a fake image.

The accuracy is defined as the proportion of correct predictions made by the model, as expressed in Equation (2):

(2) $$accuracy=\frac{TP+TN}{TP+TN+FN+FP}$$

where TP (True Positives) represents the number of images correctly classified as forgeries, TN (True Negatives) represents the number of images correctly classified as real, FN (False Negatives) represents the number of images that are incorrectly classified as real, and FP (False Positives) represents the number of images that are incorrectly classified as forgeries. These calculations are performed in the context of fake image classification. The AUC metric is calculated as the area under the ROC curve, where the ROC curve is a plot of the true positive rate (TPR) against the false positive rate (FPR). The IoU is defined as

(3) $$IoU=\frac{TP}{TP+FP+FN+\epsilon }=\frac{area\;of\;overlap}{area\;of\;union+\epsilon }$$

where TP, TN, FN, and FP are computed in the per-pixel classification result, with a small value ϵ added to prevent division by zero.

In the study [10], the pixel-wise accuracy metric is employed to assess the effectiveness of solving the segmentation problem. However, in scenarios where the background area exceeds that of the target object, the TN (true negative) term in Formula (2) heavily influences the metric value. Although pixel-wise accuracy may not be a suitable metric for detecting fake areas, it is commonly used to evaluate the performance of segmentation in the existing literature. To provide a meaningful comparison with previous studies, we also report pixel-wise accuracy in Table 1. In Table 2, we also use this metric to compare our results with those of previous studies.

### 4.3. Data Analysis and Results

Table 1 presents a comparison of segmentation and classification performance at *K* = 0 and *K* = 1 when using the proposed method versus not using our method. The segmentation performance is evaluated using IoU, while the classification performance is evaluated using AUC and accuracy at threshold values of 20% and 30%. From Table 1, we observe a substantial improvement in performance for the proposed method from *K* = 0 to *K* = 1, with the greatest improvement seen in detecting Face2Face manipulation. Using a threshold value of 20% yielded the best results when measuring classification performance using accuracy. The IoU values demonstrate that our method significantly outperforms the results obtained without using our method to detect all four types of forgery techniques. In terms of classification performance, our method outperforms the results obtained without using our method to detect four types of forgery techniques, as measured by both the AUC metric and the accuracy metric with a threshold set at 20%. The only exception is in the case of detecting FaceSwap and NeuralTextures, where our method slightly underperforms compared to the results obtained without using our method, as measured by the accuracy metric with the threshold set at 30%.

Based on Table 1, we conclude that the percentages of performance IoU improvement brought by the proposed method for detecting forgery methods DeepFakes, Face2Face, FaceSwap, and NeuralTextures are $\frac{0.600-0.264}{0.264}=127.2\%$, $\frac{0.795-0.375}{0.375}=112.8\%$, $\frac{0.516-0.254}{0.254}=103.1\%$, and $\frac{0.485-0.358}{0.358}=35.4\%$, respectively. Moreover, based on Table 1, the percentages of performance AUC improvement brought by the proposed method for detecting forgery methods DeepFakes, Face2Face, FaceSwap, and NeuralTextures are $\frac{0.750-0.539}{0.539}=39.1\%$, $\frac{0.742-0.498}{0.498}=48.9\%$, $\frac{0.503-0.493}{0.493}=2.0\%$, and $\frac{0.650-0.530}{0.530}=22.6\%$, respectively.

**Figure 8:**
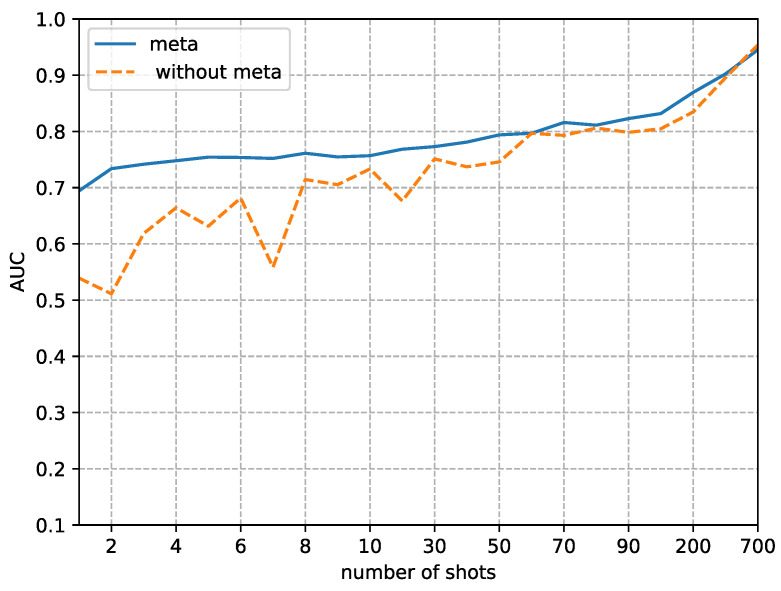
Comparison of AUC between random initial weights (without the proposed method) and meta-learning of detecting images altered by DeepFakes manipulation methods. The x-axis is the size of the fine-tuned training set and the y-axis is the value of AUC.

**Figure 9:**
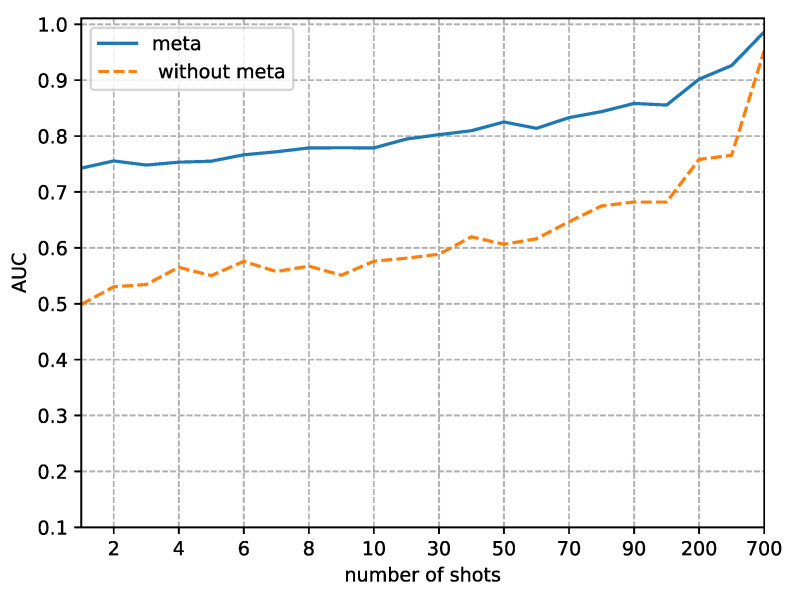
Comparison of AUC between random initial weights (without the proposed method) and meta-learning of detecting images altered by Face2Face manipulation methods. The x-axis is the size of the fine-tuned training set and the y-axis is the value of AUC.

**Figure 10:**
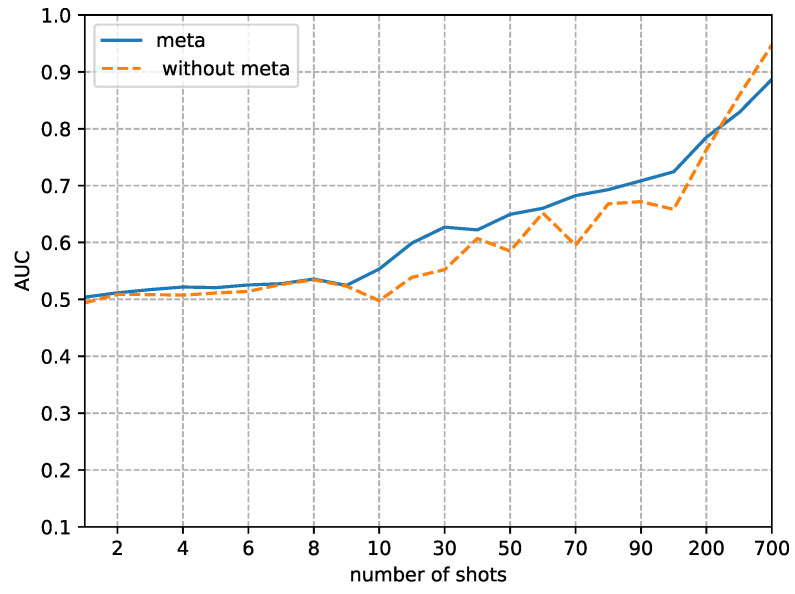
Comparison of AUC between random initial weights (without the proposed method) and meta-learning of detecting images altered by FaceSwap manipulation methods. The x-axis is the size of the fine-tuned training set and the y-axis is the value of AUC.

**Figure 11:**
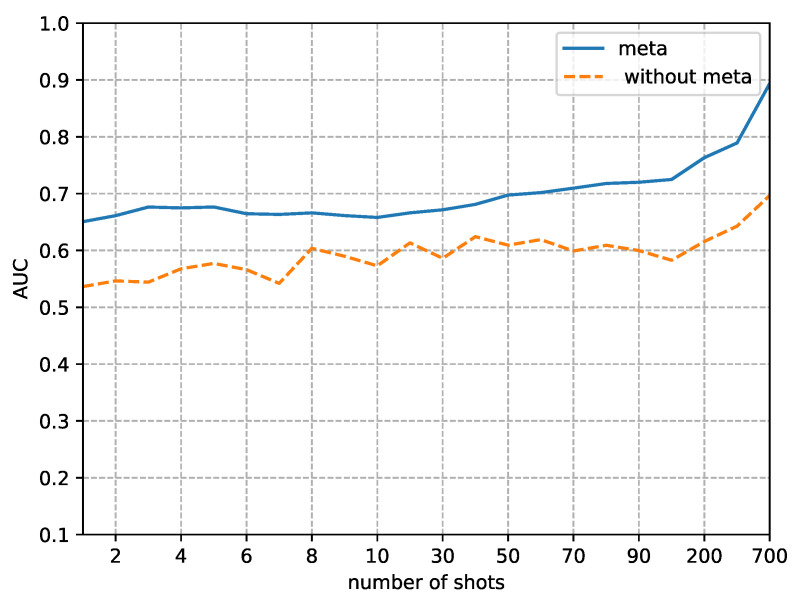
Comparison of AUC between random initial weights (without the proposed method) and meta-learning of detecting images altered by NeuralTextures manipulation methods. The x-axis is the size of the fine-tuned training set and the y-axis is the value of AUC.

Although the focus of this paper is on the performance in small sample sizes, we also analyzed the difference in detection performance between using our method and not using it as the sample size gradually increases. Figure 8, Figure 9, Figure 10 and Figure 11 respectively, demonstrate the trends in the performance changes in detecting four types of forgery techniques as the sample size gradually increases.

From Figure 8, Figure 9, Figure 10 and Figure 11, we can observe a trend where the performance of our method consistently improves as the fine-tune size *K* increases. In contrast, without using our method, the performance exhibits unstable bounce. This phenomenon is due to the random initial weights of the detector when not using our method.

The second phenomenon we observe from Figure 8, Figure 9, Figure 10 and Figure 11 is that our method’s performance is consistently superior to not using our method when the fine-tune size *K* is less than 700. When the sample size reaches 700, the performance of not using our method to detect FaceSwap and DeepFakes quickly improves, approaching the performance of our method. This result implies that the proposed method is more efficient in the case of small fine-tuned datasets than the method with random initial weights. As the size of the fine-tuned set *K* increases from 1 to 700, the advantage of the initial weights obtained by meta-learning slowly decreases.

Because our pioneering research aims to detect forgery regions and determine whether a given image is forged using small samples, the study closest to ours in published research is [10]. While their study also detects forgery regions and determines whether an input image is forged, they only experimented with two training sets and provided experimental results on the pixel-wise accuracy of forgery region detection. In Section 4.2, we explained why pixel-wise accuracy is not an appropriate metric for evaluating the detection of forgery regions. However, this paper also provides detailed results on detecting forgery regions using the pixel-wise accuracy and IoU metrics, which can serve as a reference for comparing the effectiveness of subsequent research efforts.

Table 2 compares the zero-shot result of different detection methods between [10,38] and the proposed method. In Table 2, the first two methods FT_Res and FT are proposed by Cozzolino et al. [38]. The next four methods Deeper_FT, MT_old, No_Recon, and MT_New are proposed by Nguyen et al. Based on Table 2, the proposed method has the best result in deciding the fakeness of the unseen methods DeepFakes, Face2Face, and NeuralTextures, but MT_Old has the best result in recognizing the unseen method FaceSwap.

## 5. Conclusions

This study utilized meta-learning to train a neural network that can detect fake images generated from various unseen forgery techniques with a small number of samples in contrast to the conventional approaches that train a fake image detector with a comprehensive training dataset encompassing various forgery techniques. The proposed method prioritizes utilizing the information from a small number of samples to quickly adapt the fake detector. The experimental results demonstrate that the proposed method can significantly improve the performance metrics, such as AUC, accuracy, and IoU, using a small number of samples. This also indicates that this approach is a promising direction for further research. Possible future directions include increasing the number of forgery techniques (training tasks) and improving the method of extracting features from a few samples.

This study demonstrates that the meta-learning approach enables us to train a machine capable of detecting new forgery methods from small sample sizes. Therefore, if a new forgery method emerges, it can be detected as long as a few samples are collected. As a result, in the competition with forgers, the response time of the detector can be reduced. One constraint of our approach is that it necessitates the collection of a small amount of training data. Nevertheless, considering the absence of a technique capable of identifying all novel forgery methods without additional training, obtaining a small set of training samples remains a reasonable strategy. The future research direction is to compare the impact of the number of training tasks on meta-training for detection performance.

## Figures and Tables

**Table 1:** Results for meta-learning of detecting C23 crop images altered by the unseen manipulation methods with initial weights deduced by the proposed method (inner loop iteration 1) and with *K*-shot fine-tune. In this table, *acc-pixel* stands for pixel-wise accuracy. In this table, we use the symbol *t* to denote the threshold. For instance, when t=0.3, it indicates that the threshold value is 30%. The IoUs and acc-pixels in this table are the average results for each image in the test set.

Testing	Segmentation		Classification		
Task	IoU	acc-Pixel	AUC	acc (*t* = 0.2)	acc (*t* = 0.3)
DeepFakes meta *K* = 0	0.565	0.729	0.737	0.666	0.677
DeepFakes meta *K* = 1	0.600	0.747	0.750	0.685	0.683
DeepFakes no meta *K* = 1	0.264	0.704	0.539	0.533	0.532
Face2Face meta *K* = 0	0.576	0.899	0.729	0.647	0.507
Face2Face meta *K* = 1	0.795	0.890	0.742	0.704	0.525
Face2Face no meta *K* = 1	0.375	0.388	0.498	0.499	0.500
FaceSwap meta *K* = 0	0.410	0.851	0.495	0.482	0.491
FaceSwap meta *K* = 1	0.516	0.839	0.503	0.500	0.492
FaceSwap no meta *K* = 1	0.254	0.584	0.493	0.494	0.494
NeuralTextures meta *K* = 0	0.458	0.862	0.623	0.557	0.516
NeuralTextures meta *K* = 1	0.485	0.870	0.650	0.582	0.517
NeuralTextures no meta *K* = 1	0.358	0.587	0.530	0.518	0.519

**Table 2:** Comparison of detecting images altered by the unseen manipulation methods with the proposed method and the method of Nguyen et al. [10] with zero-shot. In this table, bold font is used to emphasize the best values of each indicator. In this table, acc-pixel stands for pixel-wise accuracy. In order to shorten the length of the table, DF in this table stands for DeepFakes, FS stands for FaceSwap, F2F stands for Face2Face, and NT stands for NeuralTextures.

Manipulation	Classification (Accuracy)				Segmentation (acc-Pixel)			
Method	DF	FS	F2F	NT	DF	FS	F2F	NT
FT_Res [38]	0.647	0.535	-	-	-	-	-	-
FT [38]	0.621	0.523	-	-	-	-	-	-
Deeper_FT [10]	0.512	0.534	-	-	-	-	-	-
MT_Old [10]	0.537	**0.568**	-	-	0.701	0.842	-	-
No_Recon [10]	0.519	0.549	-	-	0.704	0.848	-	-
MT_New [10]	0.523	0.540	-	-	0.703	0.847	-	-
Proposed method	**0.666**	0.482	**0.647**	**0.557**	**0.729**	**0.851**	**0.899**	**0.862**

## Data Availability

Not applicable.
